# Complete mitochondrial genome sequence of *Lepus yarkandensis* Günther, 1875 (Lagomorpha, Leporidae): characterization and phylogenetic analysis

**DOI:** 10.3897/zookeys.1012.59035

**Published:** 2021-01-26

**Authors:** Wenjuan Shan, Mayinur Tursun, Shiyu Zhou, Yucong Zhang, Huiying Dai

**Affiliations:** 1 Xinjiang Key Laboratory of Biological Resources and Genetic Engineering, College of Life Science and Technology, Xinjiang University, Urumqi, China, 830046 Xinjiang University Urumqi China

**Keywords:** mitogenome, molecular phylogeny, synonymous/non-synonymous substitution, Yarkand hare

## Abstract

*Lepus yarkandensis* is a national second-class protected animal endemic to China and distributed only in the hot and arid Tarim Basin in Xinjiang. We sequenced and described the complete mitogenome of *L. yarkandensis* to analyze its characteristics and phylogeny. The species’ DNA is a 17,047 bp circular molecule that includes 13 protein-coding genes (PCGs), two rRNA genes, 22 tRNA genes, and one control region. The overall base composition was as follows: A, 31.50%; T, 29.40%; G, 13.30% and C, 25.80%, with a high A+T bias of 60.9%. In the PCGs, ND6 had deviation ranges for AT skew (–0.303) and GC skew (0.636). The Ka/Ks values of ND1 (1.067) and ND6 (1.352) genes were >1, indicating positive selection, which might play an important role in the adaptation of *L. yarkandensis* to arid and hot environments. The conserved sequence block, the central conserved domain, and the extended termination-associated sequences of the control region and their features were identiﬁed and described. The phylogenetic tree based on the complete mitogenome showed that *L. yarkandensis* was closely related to the sympatric *Lepus tibetanus pamirensis*. These novel datasets of *L. yarkandensis* can supply basic data for phylogenetic studies of *Lepus* spp., apart from providing essential and important resource for further genetic research and the protection of this species.

## Introduction

The Yarkand hare (*Lepus yarkandensis*) is endemic to China and is restricted to scattered oases around the Taklamakan Desert in the Tarim Basin of Xinjiang (Luo 1988; Smith et al. 2008, 2018). These hares live in hot, arid environments with scarce food and open terrain. Thus, this species is highly morphologically specialized, with smaller bodies, longer ears, and larger tympanic bullae than other *Lepus* species in China (Shan et al. 2011; Wu et al. 2011). This species is also listed as a second-class protected animal (Wang 1998). Several studies have been published on *L. yarkandensis*, including its morphology, skull morphometrics, genetic diversity, and genetic structures based on partial mitochondrial DNA (mtDNA) markers, microsatellites, and several nuclear genes (Li et al. 2005; Li et al. 2006; Aerziguli et al. 2010; Shan et al. 2011). The complete mtDNA sequence of *L. yarkandensis* has been reported (Huang et al. 2019), but without the details given of its characteristics, particularly those adapting to such extremely arid environments.

Characterized by small size, stable gene content, high evolutionary rate, relatively conserved gene arrangement, high information content, and maternal inheritance, animal mitogenomes are powerful tools used to investigate molecular evolution, phylogenetic relationships, and protective biology for many animals (Yu et al. 2017; Zhang et al. 2018; Song et al. 2019; Hu et al. 2020; Wu et al. 2020).

In the present study, we successfully sequenced and characterized the complete mtDNA of *L. yarkandensis*, including its base composition, gene structure, and arrangement of protein-coding genes (PCGs) and a control region. We also constructed a phylogenetic tree based on complete mitogenome sequences to elucidate the relationship of *L. yarkandensis* with other *Lepus* spp. Therefore, this study provides essential scientific data and contributes to population genetics, adaptation, and phylogenetic studies of *L. yarkandensis*.

## Materials and methods

A male adult *L. yarkandensis* was collected from Alar, Xinjiang, China (40°34'00"N, 81°19'33"E) on 24 December 2016. Complete mtDNA was extracted from muscle tissue using standard phenol-chloroform (Psifidi et al. 2010). The complete mitogenome of the species was sequenced by next-generation sequencing using an Illumina HiSeq platform by Hengchuang Gene Technology Co., Ltd (Shenzhen, China) and assembled using SOAPdenovo 12.04 (Luo et al. 2012). The genome structure was mapped using the CGView software (Stothard et al. 2005). The complete mitogenome sequences of 25 other lagomorph species were downloaded from GeneBank (Table 1). The base composition, Ka and Ks (Ka, Ks, Ka/Ks) values, and composition skew were analyzed using MEGA7, together with the following formulas: AT skew = [A – T]/ [A + T] and GC skew = [G – C] / [G + C] (Perna et al. 1995). A conserved sequence block (CSB) in the control region was identiﬁed based on previously published sequence data from several mammals (Sbisà et al. 1997). All tRNA secondary structures, except for tRNA-Ser (AGN), were verified using the tRNAscan-SE Webserver (Lowe and Eddy 1997). A phylogenetic tree was constructed by neighbor-joining (NJ) using MEGA7 and Bayesian analysis using MrBayes (Ronquist et al. 2012; Kumar et al. 2016). An NJ tree was constructed with default settings. Bayesian analyses were performed using MrBayes v. 3.2.6 ×64 for the best-fit model, GTR+I+F+G4, as determined by IQ-TREE (Nguyen et al. 2015). With the final model, analyses were run for 5,000,000 generations.

**Table 1.** Lagomorph mitogenomes used in the phylogenetic analysis of the present study.

Name	Accession number	Collection places	Size
*Lepus americanus1*	NC024043	Montana, USA	17042
*Lepus americanus2*	KJ397613	Montana, USA	17042
*Lepus capensis*	GU937113	Yancheng, Jiangsu	17722
*Lepus coreanus*	KF040450	Incheon, Korea	17472
*Lepus europaeus1*	AJ421471	Skane, Sweden	17734
*Lepus europaeus2*	KY211025	North-east Greece	16680
*Lepus granatensis1*	NC024042	León, Spain	16916
*Lepus granatensis2*	KJ397610	León, Spain	16916
*Lepus hainanus*	JQ219662	Hainan, China	16646
*Lepus sinensis*	KM362831	Hefei Anhui	17438
*Lepus tibetanus pamirensis*	LC073697	Aketao, Xinjiang,	17597
*Lepus timidus1*	KR019013	Haerbin, Heilongjiang	17762
*Lepus timidus2*	KJ397605	Finland	17755
*Lepus timidus3*	KR030070	Harbin, Heilongjiang	17748
*Lepus timidus4*	KR030072	Harbin, Heilongjiang	17749
*Lepus timidus5*	KR030069	Harbin, Heilongjiang	17744
*Lepus timidus6*	KR013248	Harbin, Heilongjiang	17759
*Lepus tolai*	KM609214	Hefei Anhui	17472
*Lepus townsendii1*	NC024041	Wyoming, USA	17732
*Lepus townsendii2*	KJ397609	Wyoming, USA	17732
*Lepus yarkandensis1*	MG279351	Alaer, Xinjiang	17047
*Ochotona curzoniae*	EF535828	Qinghai, China	17313
*Ochotona collaris*	AF348080	Not mentioned	16968
*Ochotona princep*s	AJ537415	Not mentioned	16481
*Lepus yarkandensis2*	MN450151	Kuqa, Xinjiang	17011
*Oryctolagus cuniculus*	AJ001588	Not mentioned	17245

## Results and discussion

### Mitochondrial genome organization

**Figure 1.**
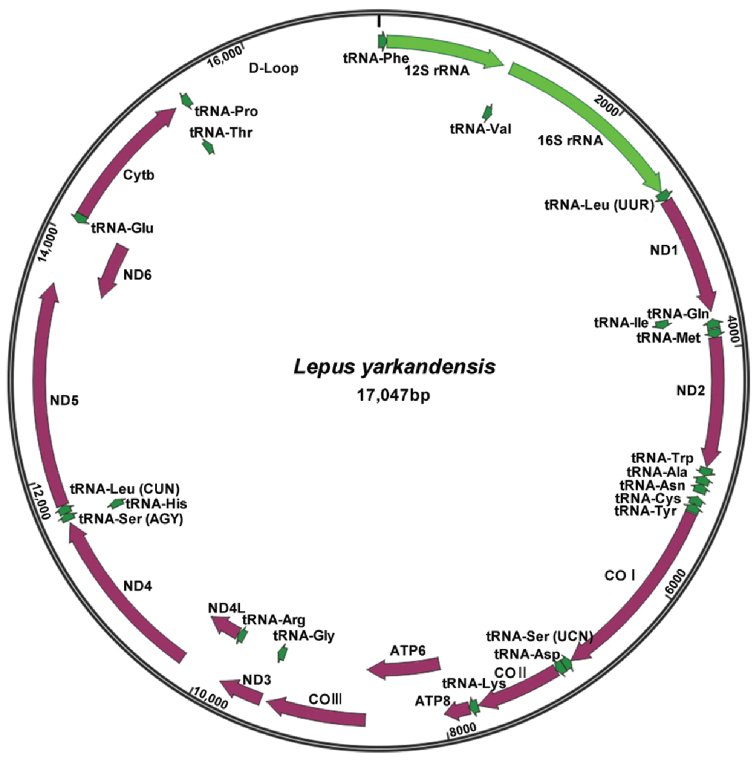
Complete mitochondrial genome map of *Lepus yarkandensis*. Genes encoded on the heavy and light strands are shown outside and inside the circle, respectively.

The mitogenome of *L. yarkandensis* was a circular, double-stranded DNA molecule 17047 bp in size (GenBank accession number: MG279351) which is slightly longer than reported *L. yarkandensis* (MN450151) with 17011 bp (Huang et al. 2019). It contained all 37 typical vertebrate mitogenomes–13 PCGs, two rRNA genes, 22 tRNA genes, and one control region-among which 28 genes were encoded on the heavy strand (H strand), except for eight tRNA genes and the ND6 gene (Fig. 1; Table 2). Eleven overlapping nucleotides with lengths ranging from 1 bp to 47 bp were present in the *L. yarkandensis* mitogenome, comprising a total length of 140 bp, with the longest nucleotide located between ND4 and tRNA-His. Moreover, 70 bp of intergenic spacer sequences spread over 12 regions in the hare mitogenome, ranging from 1 bp to 32 bp in size, with the longest was located between tRNA-Asn and tRNA-Cys (Table 2).

**Table 2.** Mitochondrial genome organization of *Lepus yarkandensis*.

Gene name	Position		Size	Location	Codon		Intergenic nucleotide bp
	From	To	(bp)	H/L strand	Start	Stop	
tRNA-Phe	1	67	67	H			0
12S rRNA	68	1022	955	H			0
tRNA-Val	1023	1088	66	H			0
16S rRNA	1087	2668	1582	H			–2
tRNA-Leu (UUR)	2669	2743	75	H			0
ND1	2746	3702	957	H	ATG	T	+2
tRNA-Ile	3701	3769	69	H			–2
tRNA-Gln	3767	3838	72	L			–3
tRNA-Met	3848	3916	69	H			+9
ND2	3917	4960	1044	H	ATT	TAA	0
tRNA-Trp	4966	5032	67	H			+5
tRNA-Ala	5035	5101	67	L			+2
tRNA-Asn	5102	5174	73	L			0
tRNA-Cys	5207	5273	67	L			+32
tRNA-Tyr	5274	5339	66	L			0
COI	5347	6888	1542	H	ATG	TAA	+7
tRNA-Ser (UCN)	6891	6959	69	L			+2
tRNA-Asp	6963	7031	69	H			+3
COII	7032	7715	684	H	ATG	TAG	0
RNA-Lys	7719	7789	71	H			+3
ATP8	7791	7994	204	H	ATG	TAA	+1
ATP6	7952	8632	681	H	ATG	TAA	–43
COIII	8632	9435	804	H	ATG	T	–1
tRNA-Gly	9416	9485	70	H			–20
ND3	9486	9842	357	H	ATT	TA	0
tRNA-Arg	9833	9899	67	H			–10
ND4L	9901	10197	297	H	ATG	TAA	+1
ND4	10191	11615	1425	H	ATG	T	–7
tRNA-His	11569	11637	69	H			–47
tRNA-Ser (AGY)	11638	11696	59	H			0
tRNA-Leu (CUN)	11697	11766	70	H			0
ND5	11767	13578	1812	H	ATT	TAA	0
ND6	13575	14099	525	L	ATG	TAG	–4
tRNA-Glu	14100	14167	68	L			0
Cytb	14171	15310	1140	H	ATG	AGG	+3
tRNA-Thr	15310	15377	68	H			–1
tRNA-Pro	15378	15443	66	L			0
D-Loop	15444	17047	1604	H			0

(Overlap is denoted as “–”. Spacer regions are denoted as “+”. No overlap or interval is denoted as “0”.)

### Genome composition and skewness

AT skew, GC skew, and A + T content were selected as parameters for investigating the pattern of the mitogenome nucleotide composition (Wei et al. 2010; Hassanin et al. 2005). The *L. yarkandensis* mitogenome had a base nucleotide composition of 31.50% for A, 29.40% for T, 13.30% for G, and 25.80% for C, with an A+T bias of 60.90%. Moreover, A and C were more popular than T and G with overall AT skew = 0.034 and GC skew = –0.320 in the entire *L. yarkandensis* mitogenome (Table 3). These overall genome composition and skewness are highly similar to those of other *Lepus* spp., such as *L. yarkandensis* (MN450151), *Lepus coreanus* and *Lepus tolai* (Yu et al. 2015; Huang et al. 2019; Shan et al. 2020). However, in species such as *Caenorhabditis elegans*, *Ascaris suum*, and *Mytilus edulis*, different AT and GC skew values were determined-negative AT skew and positive GC skew (Perna et al. 1995). In *Arbacia lixula* and *Anopheles cracens*, both AT and GC skews were negative (Perna et al. 1995; Mao et al. 2019). Moreover, an AT-rich region is typically observed in vertebrates (Quinn et al. 1993; Zhao et al. 2016; Sarvani et al. 2018). Thus, this variation in AT and GC skews shows a degree of similarity within the same genus but not in different classes, which can also be used as an auxiliary reference for evaluating phylogenetic relationships.

**Table 3.** Nucleotide composition and skewness of the *Lepus yarkandensis* mitogenome.

	A%	T%	G%	C%	Size	A+T%	ATskew	GCskew
Total PCGs	30.50	30.90	12.00	26.50	11417	61.40	–0.007	–0.377
Overall	31.50	29.40	13.30	25.80	17047	60.90	0.034	–0.320
rRNAs	36.10	24.70	17.80	21.40	2535	60.80	0.188	–0.092
tRNAs	31.20	29.90	12.30	26.70	8295	61.10	0.021	–0.369
D-Loop	28.70	27.40	13.00	30.90	1604	56.10	0.023	–0.408
CDs	21.80	27.10	21.10	30.0	317	48.90	-0.108	–0.174
CSB	30.00	26.2	11.4	32.4	920	56.2	0.068	–0.480
ETAS	31.60	30.80	9.80	27.80	367	62.40	0.013	–0.479

### Protein-coding genes

The total length of PCGs in the *L. yarkandensis* mitogenome was 11,417 bp, and its base composition was 30.50% for A, 30.90% for T, 12.00% for G, and 26.50% for C with an A+T bias of 61.40%. Among the 13 PCGs, 12 were located on the heavy strand (H strand), whereas ND6 was located on the light strand (Tables 2, 3), as observed in other *Lepus* species (Ding et al. 2014; Shan et al. 2020).

The skewness of the entire PCGs in *L. yarkandensis* (Table 3) indicated a higher occurrence of T than A, with a negative AT skew (–0.007), and C than G with a negative GC skew (–0.337) (Table 3). The negative AT skew value was inconsistent with that for most mammalians, which had positive AT skew values (Sarvani et al. 2018; Priyono et al. 2020). However, the result of the current study is highly similar to the result obtained for *Camelus dromedarius* (both AT and GC skews were negative), a heat-tolerant mammal (Sarvani et al. 2018; Manee et al. 2019).

**Figure 2.**
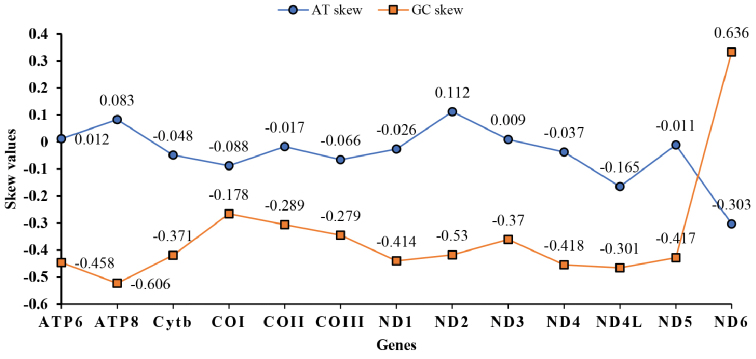
GC and AT skews for mitochondrial PCGs in *Lepus yarkandensis*.

To further estimate and understand the level of base bias between all PCGs, we calculated the AT and GC skew ratios for each PCG in the mtDNA genome of *L. yarkandensis* (Fig. 2). All values for the skewness of GC (except for ND6) in PCGs were negative, with C being more prevalent that G in the nucleotide composition. The ATP6, ATP8, ND2, and ND3 genes had positive AT skews, whereas the remaining genes (9 of 13) had negative values. Notably, ND6 had deviation ranges for AT skew (–0.303) and GC skew (0.636) when compared with the other 12 PCGs in the *L. yarkandensis*mtDNA sequence, and the deviation range is highly similar to some mammalians, such as *Moschiola indica*, *Camelus dromedarius*, and *Bubalus quarlesi* (Sarvani et al. 2018; Manee et al. 2019; Priyono et al. 2020).

As with the vertebrate mtDNA genome, the majority of PCGs in the *L. yarkandensis* mitogenome used ATG as the start codon, although ND2, ND3, and ND5 used ATT as the start codon. Most PCGs used typical stop codons (TAA for ND2, COI, COII, ATP8, ATP6, ND4L, and ND5; TAG for ND6 and COII), whereas a small number of abnormal stop codons were observed, including AGG (Cytb), T (ND1, COIII, ND4), and TA (ND3). Moreover, nine of 13 PCGs had complete stop codons, and four genes had incomplete stop codons (Table 2), which could be completed via posttranscriptional polyadenylation (Anderson et al. 1981; Ojala et al. 1981). Both PCGs of our *L. yarkandensis* (MG279351) and reported Yarkand hare (MN450151) have identical start and end codons, but different skewness.

**Figure 3.**
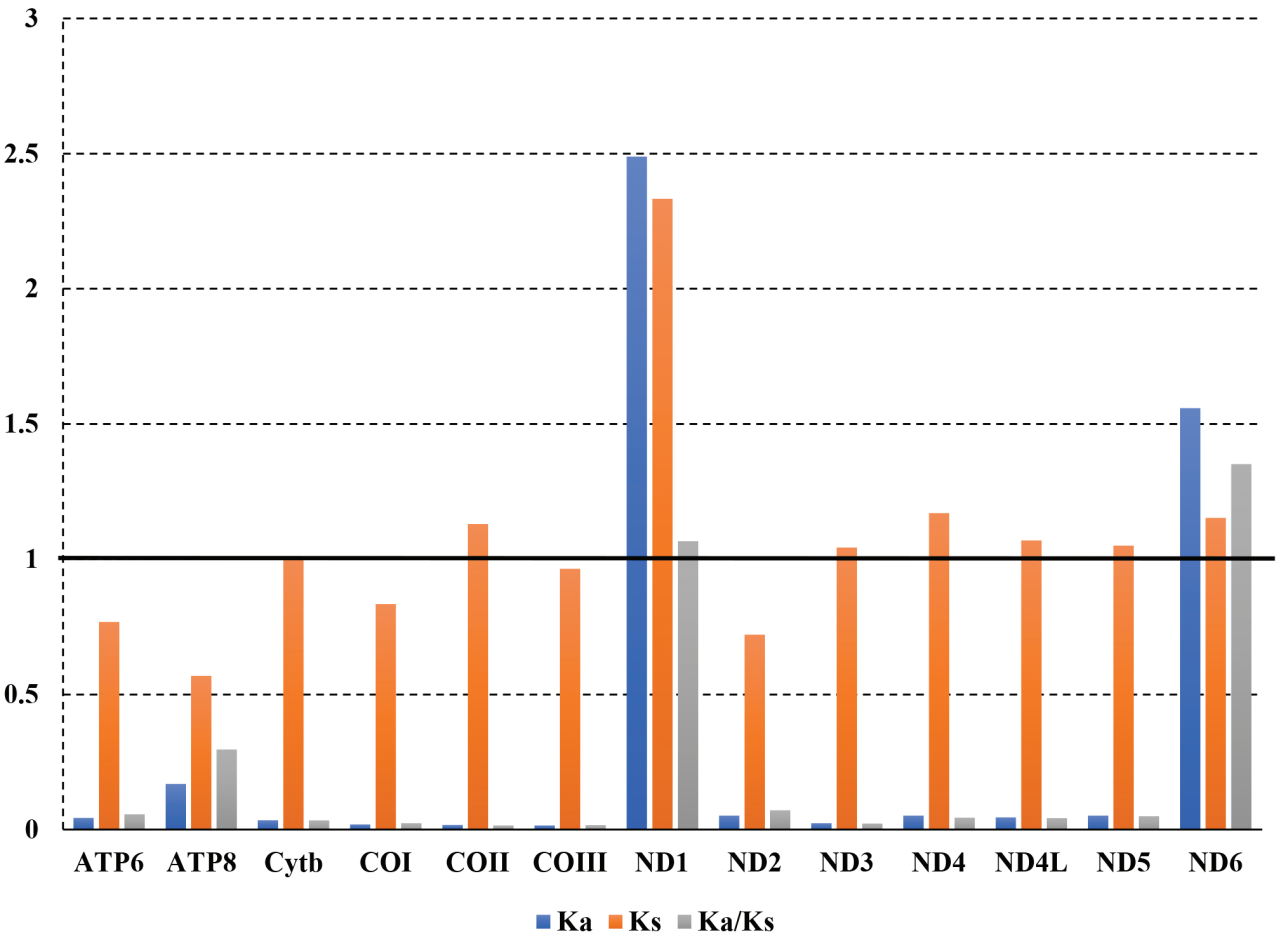
Evolutionary rates of the *Lepus yarkandensis* mitogenome by Ka/Ks.

The Ka, Ks, and Ka/Ks values of PCGs were estimated using substitution rates (Fig. 3). If Ka/Ks > 1, a positive selection effect was considered; if Ka/Ks = 1, a neutral effect was assumed; and if Ka/Ks < 1, purification selection was considered (Hurst et al. 2002). Except for ND1 and ND6, all PCGs in *L. yarkandensis* had average Ka/Ks values < 1, indicating purification selection. Meanwhile, for ND1 and ND6, Ka/Ks > 1 indicated positive selection. The function of the mitochondrial genome is crucial because it mainly undergoes evolutionary neutral or purifying selection. Other studies have reported that mitochondrial genes are also influenced by positive selection, particularly in animals adapting to harsh environments (Luo et al. 2008; Hichem et al. 2017; Jin et al. 2018). In the present study, positive selection in ND1 and ND6 might be beneficial to organisms and may confer to *L. yarkandensis* the ability to adapt to harsh and arid environments.

### Control region

**Figure 4.**
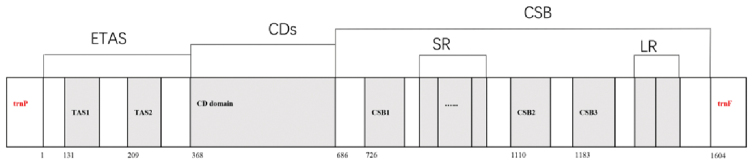
A schematic of the structural organization of the mitochondrial control region in *Lepus yarkandensis*. Control region ﬂanking genes tRNA-Phe and tRNA-Pro presented in red. Conserved elements in the control region denoted by gray boxes: TAS, termination associated sequence; CD, central conserved domain; CSB, conserved sequence block. SR, short repeat; LR, long repeat.

The control region 1604 bp in length was organized between *trnP* and *trnF* genes in the *L. yarkandensis* mitogenome (Table 2; Fig. 4). In vertebrate mitogenomes, the control region is a noncoding segment and consists of several control elements. These elements regulate genome replication and transcription (Boore 1999). In the current study, we successfully identified several highly conserved domains within the control region of the *L. yarkandensis* mitogenome-conserved sequence blocks (CSB) I–III, conserved domain (CD), and extended termination associated sequence (ETAS) I–II–on the basis of their homology with other members of Lagomorpha and mammals (Elisabetta et al. 1997) (Table 4; Fig. 4). Characteristic motifs were used to detect the CSB domains: CSBI (GACATA), CSBII (CAAACCCCCC), and CSBIII (TGCCAAACCCCAAAAAC) (Gemmell et al. 1996; Elisabetta et al. 1997). We found from the sequence alignment results among hares and other mammals (Elisabetta et al. 1997) that more variations existed in Yarkand hare, including base insertions and deletions in the whole control region. CD was conservative with a narrow length range. The ETAS and CSB regions widely varied in the length of the control region, which is also the main reason for variations in mitogenome size in different species (Xu et al. 2012).

In CSB regions, CSB1 and CSB3 were relatively conservative, and CSB2 widely varied in *L. yarkandensis*. This finding contradicted the results for *Felis catus* and Mustelidae species (Elisabetta et al. 1997l; Zhang et al. 2009). In the present study, an ACCCC motif in the ETAS I sequence of *L. yarkandensis* was found, similar to that of the horseshoe bat (Sun et al. 2009). In some taxa such as species of Mustelidae, cattle, and Cervidae, the sequences were GCCCC (Zhang et al. 2009; Douzary et al. 1997). Between CSB I and CSB II, a number of short tandem repeat motifs, which commonly characterize mitogenomes, were observed in the *L. yarkandensis* mitogenome (Ren et al. 2009). The short repeat CGTCTACGCGCACGTACACCCA was 22 bp with 14 repetitions (Table 4), whereas the long repeat ACAATACTGACATAGCACTCAGCCTTTTATTTTTCCTCCAACAGGCATAACCCTAATTAAATTTTTCCAAAAAAAA occurred twice. Similarly, the short repeats CSB3 CGTCTACGCGCACGTACACCCA in *L. yarkandensis* (Fig. 4) occurred twice, which was also found in other *Lepus* species in this study. Notably, tandem repeats have been described in the control region of metazoans (Lunt et al. 1998; Rand et al. 1993; Yokobori et al. 2004) and the family Veneridae.

**Table 4.** Sequences of the conserved regions in the control region of *Lepus yarkandensis*.

Functional domains	Nucleotide sequences
TAS	
ETAS1	ACCATTATATGTTTAATCGTACATTAAAGCTTTACCCCATGCATATAAGCTAGTACATTC
ETAS2	CACATACACCTACTCAACTCCACAAAACCTTATCATCAACACGGATATCCAAACCCATTACCCA
CSB	
CSB1	TATCTTTTCATGCTTGACGGACATA
CSB2	AAACCCCCCCTACCCCC
CSB3	TGCCAAACCCCAAAAAC

### Transfer RNAs and ribosomal RNAs

Except for tRNA-ser (AGY), which lacked a D stem, the other 21 tRNAs formed complete secondary structures (Suppl. material 1). Aberrant loops have been found in some tRNA genes. These mismatches could be rectified by the post-transcriptional RNA-editing mechanism to maintain tRNA functions (Tomita et al. 2002).

### Phylogenetic analysis

**Figure 5.**
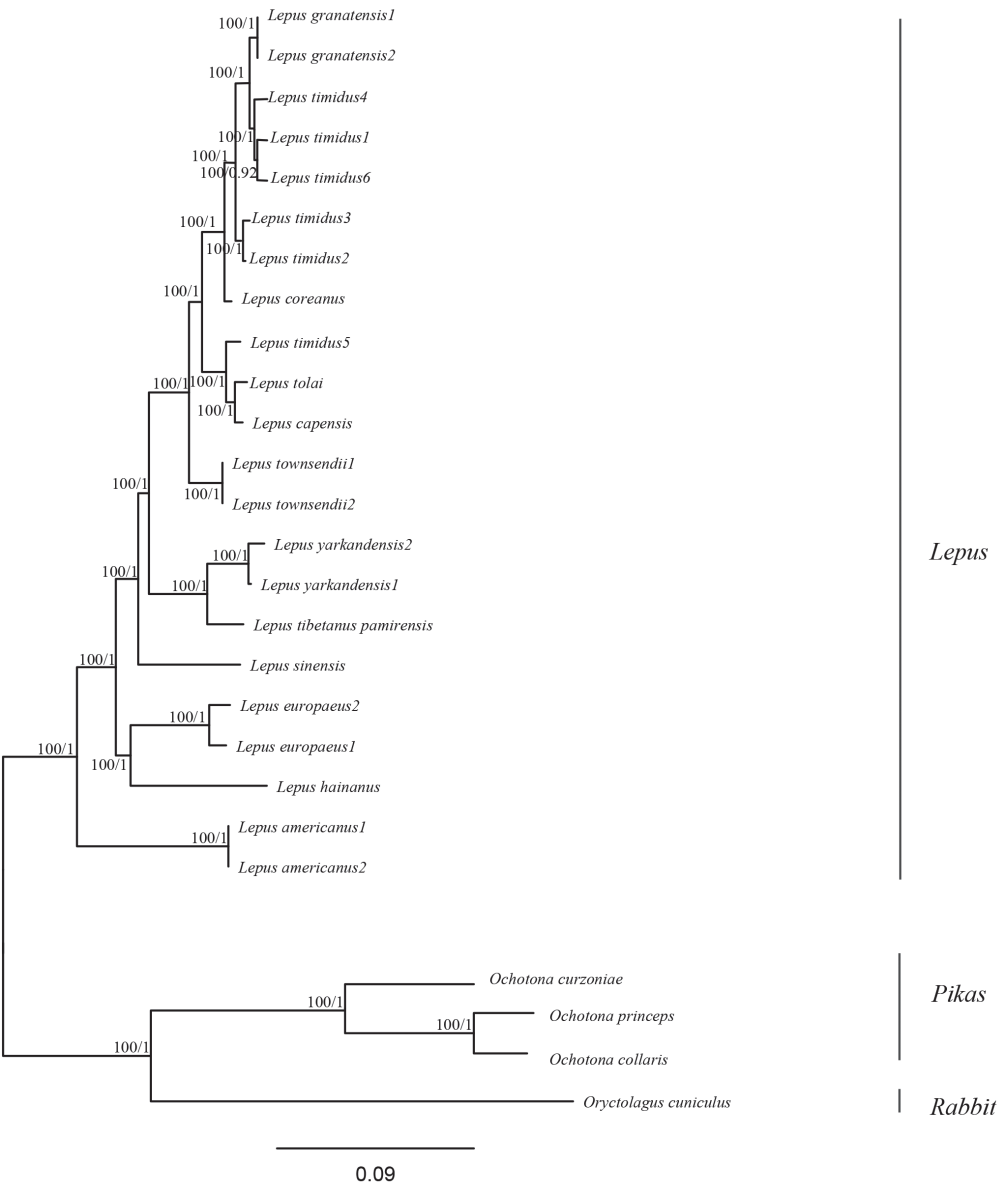
Neighbor-joining and Bayes trees based on the complete mtDNA sequences of 25 lagomorphs. Values separated by slash (/) represent bootstrap support values for the NJ and Bayes trees.

We constructed NJ and Bayesian trees based on the complete mtDNA genome of *L. yarkandensis* in this study and 25 other lagomorphs published on NCBI (Fig. 5). The topological structures of both trees were consistent and supported by high bootstrap values. The phylogenetic tree confirmed the existence of three distinct lineages-hares, rabbits, and pikas-which is consistent with Smith et al. (2018). In the present study, *L. yarkandensis* was not closely related to neither *Lepus europaeus* nor *Lepus americanus* but was closely related to *Lepus tibetanus* in Xinjiang, China. The latter was misnamed as *Lepus capensis pamirs* in our previous study (Shan et al. 2015) and was renamed by Smith et al. (2018). Our *L. yarkandensis and L. yarkandensis* (MN450151) were clustered on the same branch. One reason for this close relationship could be the relatively close habitat. *Lepus t. pamirensis* are mainly distributed in the Pamir plateau of southeastern Kashgar, Xinjiang, China, bordering the Tarim Basin. The *L. yarkandensis* sample used in the current study was from Alar City in western Tarim Basin, which is near the *L. t. pamirensis* distribution. Another reason could be similarly extreme environments. Both habitats are dry with scarce rainfall and a lack of food (Shan et al. 2011). However, the phylogenetic relationship between *L. yarkandensis* and *L. t. pamirensis* remains uncertain, as hybridization has occurred between them (Wu et al. 2011). Further analysis with more samples and more extensive markers is required.

## Supplementary materials

Supplementary material 1 Figure S1a, S1b https://doi.org/10.3897/zookeys.1012.59035.suppl1 Data type multimedia https://binary.pensoft.net/file/501677This dataset is made available under the Open Database License (http://opendatacommons.org/licenses/odbl/1.0/). The Open Database License (ODbL) is a license agreement intended to allow users to freely share, modify, and use this Dataset while maintaining this same freedom for others, provided that the original source and author(s) are credited.Wenjuan Shan, Mayinur Tursun, Shiyu Zhou, Yucong Zhang, Huiying Dai
